# 

**Published:** 1915-08

**Authors:** 


### CONTENTS.

ORIGINAL COMMUNICATIONS—

Acute Intestinal Obstruction: Notes on the Diagnosis and Treatment, by W. C. Gewin, M. D. ........ 209

Pellegra, by Geo. C. Mizell ........ 214

Observation on Syphilitic Disease of the Nervous System, by Lewis M. Gaines, M. D., Atlanta, Ga.... 221

Major Operations Under Local Anesthesia—With Report of Cases, by W. A. Selman, M. D. ........ 225

The Harrison Anti-Narcotic Law, by Mr. Hooper Alexander, U. S. District Attorney for the Northern District of Georgia, Atlanta, Ga. ........ 230

EDITORIAL ........ 234

SELECTIONS AND ABSTRACTS ........ 239

